# Ductal Carcinoma In Situ: What Can We Learn from Clinical Trials?

**DOI:** 10.1155/2012/296829

**Published:** 2012-05-09

**Authors:** Lucio Fortunato, Igor Poccia, Ugo de Paula, Elena Santini

**Affiliations:** ^1^Department of Surgery, Senology Unit, San Giovanni Addolorata Hospital, Via Amba-Aradam 9, 00184 Rome, Italy; ^2^Division of Plastic and Reconstructive Surgery, University Campus Bio-Medico, 00128 Rome, Italy; ^3^Department of Radiotherapy, San Giovanni Addolorata Hospital, 00184 Rome, Italy; ^4^Department of Radiology, San Giovanni Addolorata Hospital, 00184 Rome, Italy

## Abstract

Ductal Carcinoma in situ has been diagnosed more frequently in the last few years and now accounts for approximately one-fourth of all treated breast cancers. Traditionally, this disease has been treated with total mastectomy, but conservative surgery has become increasingly used in the absence of unfavourable clinical conditions, if a negative excision margin can be achieved. It is controversial whether subgroups of patients with favourable in situ tumors could be managed by conservative surgery alone, without radiation. As the disease is diagnosed more frequently in younger patients, these issues are very relevant, and much research has focused on this topic in the last two decades. We reviewed randomized trials regarding adjuvant radiation after breast-conservative surgery and compared data with available retrospective studies.

## 1. Introduction

Ductal carcinoma in situ (DCIS) is the fourth leading cancer among women in the United States, and its incidence has dramatically increased since the introduction of screening mammography. In the last 30 years we have witnessed a fourfold increase in its incidence [1], so that approximately one-fifth of all screen detected breast cancers are diagnosed as DCIS [2]. The relevance of this problem is evidenced by the large amount of data available in the literature, with more than 10,000 articles published on the issue, so far [3].

Interestingly, while the incidence of DCIS is increasing at a 15% annual rate in all age groups, the incidence of invasive breast cancer has been decreasing along with overall mortality for breast cancer [4].

Standard of care for surgical treatment of DCIS has long been represented by total mastectomy, with cure rates approaching 100%. Mastectomy remains indicated if the disease is too extensive to be resected with a good cosmetic outcome, in the case of inability to achieve negative margins, in the case of micropapillary DCIS or DCIS with nipple discharge, or if there are contraindications to radiotherapy (RT) in high-risk patients.

However, as breast conservation has been demonstrated equivalent to mastectomy for breast cancer patients in terms of overall survival in 6 prospective-randomized trials [5], conservative approaches have become appealing for this preinvasive disease.

It is evident that the search for the appropriate surgical treatment of DCIS is strategic, because while the disease is often multifocal, approximately 40% of recurrences are invasive [6]. Additionally, DCIS is often diagnosed in women during the “active” years of their lives, as approximately half of them become aware of this disease before the age of 60 [7], and roughly one-third before the age of 50 [3]. Therefore, while prevention of LR is a major goal of the treatment of DCIS if one wishes to maintain this disease always curative, the need to maintain a good body image for these patients cannot be understressed.

As our knowledge and understanding of DCIS has evolved in the last decades, the treatment decision-making process has become increasingly complex, and it remains one of the most controversial aspects in breast cancer treatment. This is well evidenced by a recent report on treatment of DCIS in the United States, describing that at the present time 30% of women with DCIS are treated with mastectomy, 40% with conservative surgery plus RT, and 30% with excision alone [8]. Additionally, data on treatment trends for DCIS in the USA have documented a shift in the last 15 years with a decrease of mastectomy in favour of breast conservation plus RT [3].

Breast conservation for DCIS is an issue of particular relevance and interest, because it is well documented that, on the other hand, mastectomy rates are on the rise in the USA as in other parts of the world [9]. Furthermore, a threefold increase of contralateral “prophylactic” mastectomy has been reported in the last decade [10], and it is recognized that DCIS is a marker for increased risk of invasive carcinoma in both breasts [11].

Several clinical factors may help to explain the tendency to implement this approach, including a more liberal use of MRI in the last few years with appreciation that the disease may be more extensive than previously recognized [12], and adoption of “conservative” mastectomies with immediate reconstruction, including nipple-sparing approaches.

The aim of the present report is to critically analyze data from randomized trials for DCIS and to compare their conclusions with data from retrospective series.

## 2. Randomized Trials

Five prospective randomized trials were reported in the last two decades regarding adjuvant treatment after surgery for DCIS. Four of them focused on the need of adjuvant RT after conservative surgery (Table 1), while two also investigated the addition of tamoxifen to lumpectomy plus RT.

### 2.1. NSABP B-17

In NSABP B-17 [13] 818 women with DCIS were randomized to undergo either lumpectomy only (*n* = 403) or lumpectomy followed by breast irradiation (*n* = 410) to a total dose of 50 Gy (9% of patients received 10 Gy boost to the tumor bed). Histologically negative surgical margins were required in both groups, however inking of margins was not routinely used. The five-year outcomes were first reported in 1993 [14] showing a 60% lower risk of ipsilateral breast tumor recurrence for patients who received RT. Subsequent updates continued to demonstrate a large benefit for lumpectomy plus radiotherapy compared with lumpectomy [15]. The 12-year local recurrence rate was 15.7% among women who underwent breast irradiation and 31.7% among women treated by lumpectomy alone (*P* = 0.000005).

### 2.2. EORTC 10853

In a similar trial from EORTC [16] 1010 women with DCIS were randomized to undergo either lumpectomy only (*n* = 503) or lumpectomy followed by breast irradiation (*n* = 507) to a total dose of 50 Gy. Histological negative surgical margins were required in both groups. However, at central pathology review margins were close (<1 mm) or involved in 8.5% of patients. At 10 years, patients treated with local excision alone had a LR rate of 26%, compared with 15% of LR rate in the group of excision plus RT (*P* < 0.0001).

### 2.3. SWE-DCIS

In the SweDCIS trial [17] 1067 women were randomly assigned to excision plus RT (*n* = 526) or excision only (*n* = 520). RT was administered continuously with 50 Gy in 25 fractions, or as a split course treatment with 54 Gy in two series with a gap of two weeks. No boost to the tumor bed was ever used. Microscopically clear margins were not mandatory. After a median followup of 5.2 years the cumulative incidence of LR was 22% in the control group and 7% in the RT group. No differences were observed in the two groups for metastases or breast cancer deaths.

### 2.4. UK/ANZ

In the UK/ANZ trial 1701 patients were randomly assigned to RT, and/or tamoxifen (TAM), using a two by two factorial design between 1990 and 1998 [18]. This created four subgroups: excision alone, excision plus RT, excision plus TAM, and excision plus RT plus TAM. At a median followup of 4.4 years the rate of local recurrence was 5.6% in the group with RT and 13.6% in the group without RT (*P* < 0.0001). Rate of LR was not found to be significant if tamoxifen was added to RT.

### 2.5. NSABP B-24

Another large randomized study (NSABP B-24) [13] investigated the addition of tamoxifen to lumpectomy plus radiotherapy in a cohort of 1804 patients treated between 1991 and 1994. The 7-year LR rate was 11.1% in the group treated with placebo and 7.7% in the group with tamoxifen (*P* = 0.02). This represented a 31% reduction in the risk of ipsilateral breast recurrences. Tamoxifen also accounted for a 53% risk reduction of contralateral breast tumors. The 7-year OS was 95% in both groups, with a no significant increase in the incidence of endometrial cancer in the tamoxifen group (0.78% to 0.33%; *P* = 0.38).

## 3. Meta-Analysis

A recent meta-analysis of the four randomized trials on radiation therapy for DCIS [19] showed that the LR rates were 4.79% (82/1711) and 11.3% (221/1954) for the RT and the observation arm, respectively.

The analysis showed a 60% reduction of risk of invasive and DCIS ipsilateral breast cancer with adjuvant RT.

While the incidence of ipsilateral breast cancer recurrence did not differ in the two groups, there were more invasive ipsilateral breast cancers in the observation group (8.1%) compared to the RT arm (3.8%).

The likelihood of contralateral breast cancer was 1.53-fold higher (3.85% versus 2.5%, *P* = 0.03) in RT arm.

The meta-analysis was not able to identify a subgroup of women who would not need RT, although the absolute magnitude of benefit was greater in the groups at higher risk for local failure, such as young patients and those with clinically evident lesions.

Another recent meta-analysis by the Early Breast Cancer Trialists' Collaborative Group (EBCTCG) analyzed individual patient data for all four randomized trials [20]. A total of 3729 remained eligible for the analysis after the exclusion of patients with microinvasion, invasion, Paget's disease, or another cancer present at the time of initial diagnosis. Radiotherapy reduced the absolute 10-year risk of any ipsilateral breast event by 15.2% (*P* < 0.00001), and it was effective regardless of the age at diagnosis, use of tamoxifen, margin status, focality, grade, comedonecrosis, architecture, or tumor size.

Furthermore, the proportional reduction in ipsilateral breast events was greater in older than in younger women but did not differ significantly according to any other available factor.

Even for women with negative margins and small low-grade tumors, the absolute reduction in the 10-year risk of ipsilateral breast events was 18.0%  (2*P* = 0.002). After 10 years of followup, there was, however, no significant effect on breast cancer mortality, mortality from causes other than breast cancer, or all-cause mortality.

## 4. Discussion

Results of these important randomized trials have been used to justify RT for all women with this disease.

Of course, RT is time consuming for the patients, may be responsible for several local side effects, and its avoidance would be desirable in patients with a low risk of recurrence. Additionally, it is well recognized that RT is associated with higher rates of complications if a mastectomy and breast reconstruction are needed in case of relapse [21, 22].

Relapse after treatment of DCIS is not rare, and a 9.8% incidence of invasive ipsilateral second events was reported in a recent analysis of 3046 patients from the Cancer Registry of Norway, with a median followup of 5 years [23]. An analysis of the outcome of 150 patients with LR after treatment for DCIS showed that 63 of them were invasive, and that the risk of death from breast cancer was 12% in that group [24].

There are several problems regarding the design of these four randomized trials. Pathologic factors affecting local control were largely unrecognized when these studies were designed and initiated.

Roughly 70% of women were randomized and treated before 1995, an era when both diagnosis and treatment was very different from current standards. Wide free margins and mammography of the excised breast tissue are now standard practice among dedicated surgeons, and whenever a positive surgical margin is found at final pathology, a reexcision is usually recommended. Neither mammography of the surgical specimen to confirm excision of all micro-calcification, nor negative margins of excision were a mandatory achievement in three of the four trials. Furthermore, MRI was not an option for these women, and therefore patients were included in the study even if radiologic evidence of multifocality by current standards could not be excluded. Finally, we have entered an era of increasing awareness among women and diagnosis of smaller tumors is more often reported. Outcome of screening-detected DCIS treated with excision alone may be more favourable, and although recurrence rates of 15% at 5 years are reported, these are often successfully salvaged with breast conservation, with overall breast-specific survival of 99% [25].

In NSABP B-17 histologically negative surgical margins were required. However inking of margins was not routinely used and in 13% of cases margins were either involved or unknown. NSABP B-24 allowed entry of women with involved tumor margins and women whose mammograms showed residual calcifications as long as they were considered not suggestive of invasive cancer. In fact, in this trial approximately one quarter of patients had either involved or uncertain tumor margin status. In the EORTC trial, central pathology review of margins showed that they were close (<1 mm) or involved in 16% of patients. In the Swe-DCIS trial microscopically clear margins were not mandatory, and approximately 10% of patients had pathologically involved margins.

Furthermore, in NSABP B17 both DCIS and lobular carcinoma in situ were considered eligible. Treatment of lobular carcinoma in situ is very different today, as we recognize it as a different pathological entity from DCIS, with different risks of relapse and issues for local control, at least for the low- and intermediate-grade varieties.

These problems limit the ability to apply the results of these trials to patients who undergo what is now considered optimal surgery with total pathologic evaluation. In fact patients with positive margins appeared to benefit the most from adjuvant radiation therapy, and this has been proposed as a possible explanation for the differences between these trials and other retrospective experience [26].

Furthermore, preoperative evaluation of the extent of disease is changing, an important issue given the finding of the UK Sloan Project that in 30% of patients undergoing BCS for DCIS preoperative imaging underestimates the extent of disease, resulting in a requirement for further treatment [27].

Silverstein reported his experience and found that in patients without RT, and with a careful pathological evaluation and achievement of negative margins of almost 1 cm in all direction, LR rates were comparable to those of patients treated with RT in those randomized trials [28].

In a classic work from Silverstein [28] the 10-year actuarial LR rates after BCS with or without RT were 20% and 28%, respectively (*P* = 0.06). It is noteworthy that more patients had close (<1 mm) margins in the RT group compared to the group treated with excision alone (35% versus 19%).

In this study tumor size, nuclear grade, margin width, comedonecrosis and patient age were predictors of local recurrence. This finding is very similar to a recent meta-analysis involving 44 studies on the tumor characteristics as predictors of local recurrence after treatment of DCIS [29].

Silverstein incorporated these predictors in a prognostic index called the Van Nuys Prognostic Index (USC/VNPI), designed as a scoring system to support patients and clinicians regarding the need for adjuvant RT [28]. It was suggested that patients with low USC/VNPI scores (4 to 6) could be treated with excision alone, while patients with intermediate scores (7 to 9) showed an average of 10 to 15% LR benefits with the addition of RT after BCS. Patients with high score (>10) seem to be unsuitable for BCS (as recurrences are high with or without RT) and should be considered for total mastectomy.

Unfortunately, this scoring system has never been validated by prospective studies, and some feel that although the authors demonstrate a high level of dedication and expertise in the treatment of this disease, reproducibility in clinical practice has never been demonstrated.

In an attempt to clarify this controversy and to assess the role of documented free margins and the reproducibility of Silverstein's experience, the Eastern Cooperative Oncology Group (ECOG) designed a prospective single arm study of excision alone in 671 selected patients with DCIS whose diameter was less than 2.5 cm if low-grade, or less than 1 cm if high-grade [30]. Surgical specimens were sequentially sectioned and completely embedded to determine tumor size, grade, and the margin status, stratifying patients on tumor grade and intention to administer tamoxifen. Postexcision magnification mammography was also always performed to document removal of all calcifications.

In the high-grade DCIS the rate of ipsilateral recurrence was 15.3%, suggesting excision alone is associated with a high risk of local recurrence for high-grade DCIS, even if wide (almost 3 mm) surgical margins are required. In the low-/intermediate-grade group however, the 5-year rate of ipsilateral breast events was 6.1%, a result which could be considered acceptable by many patients and physicians. Results were even more favourable for tumors less than 10 mm in diameter, or for patients >45 years of age.

However, it is well recognized that while local relapse after treatment of low-grade DCIS may take longer to present clinically compared to high-grade lesions, its incidence definitely approaches the latter group with longer follow-up [31]. Therefore, interpretation of this data necessitates caution, and more time is needed to confirm the finding of this trial.

Another prospective study on 158 patients with predominantly grade 1 or 2 lesions treated exclusively with surgery without RT [32], was recently presented by Wong et al. The protocol required a margin width of 1 cm or more. The 5-year local recurrence rate was 12%, and 31% of the recurrences were invasive, resulting in premature closure of the study.

A study of women with low-grade DCIS treated with biopsy alone from 1950 to 1968 and observed for a long period of time is not only of historical interest. Even this study showed a risk of developing invasive cancer for ipsilateral relapse in 30% of cases at 15 years [33].

Recently, a long-term follow-up evaluating invasive ipsilateral breast recurrence among participants in the two NABP trials (NSABP B17 and B24) has been published [34]. This is an important study because these two trials remain the largest prospective evaluation of breast conservation for DCIS to date. While 54% of local failures were invasive, the 15-year risk of this event was 19.4% for local excision, and 8.9% for local excision plus radiation. Compared with women aged 65 years and older, women younger than 45 years showed a 2.1-fold increase of invasive recurrences. Similarly, the margin status in NSABP B24 was associated with an approximately twofold increase of invasive recurrence. Overall, breast-cancer mortality did not differ between patients who received RT and those who did not. However, women who developed an ipsilateral invasive recurrence had a 1.75-fold greater risk of death compared with those who did not, and a 7.06-fold greater risk of breast cancer-related deaths.

Therefore, the main issue in treating DCIS is the prevention of invasive relapses, because not all of them will be curable. No doubts, our attention is directed towards the identification of subgroups of patients (e.g., those with low grade tumors, age >60, adequate margins) that could avoid RT after surgical treatment of DCIS, and in the last 2 years many reports and Consensus statements have focused on this, including the Saint Gallen Consensus Conference [35], the Newport Consensus Conference III [36], and the National Consensus Cancer Network [37].

However, from a very practical point of view, even the width of surgical margin remains controversial for the treatment of DCIS, and while the “Consensus on DCIS of Philadelphia” has proposed a 10 mm margin [38], others have proposed margins of 1 to 3 mm as adequate [39].

Another controversial issue regarding DCIS is whether patients with DCIS benefit from tamoxifen. While it is well known that women with hormone-receptor positive invasive breast cancer benefit from the addition of tamoxifen, its role on local control after excision of DCIS is not well quantified.

In NSABP-B-24 trial [13], the addition of tamoxifen resulted in a risk reduction of 16% compared with RT + placebo (HR = 0.84, 95% CI = 0.60 to 1.19, *P* = 0.33), while the UKCCCR trial [18] found a nonsignificant effect in regard of all breast events.

The different findings in these two trials may be the result of differences in the patient populations: in NSABP B-24 there was a higher proportion of young patients, ER positive, and low-grade DCIS with respect to those of UKCCCR. Furthermore, NSABP B-24 included patients with positive margins. This could in part explain the discrepancies in the outcomes between the group with tamoxifen and the group with placebo.

Therefore, the question remains of whether there are subgroups of patients for whom RT or tamoxifen is more or less effective in terms of absolute risk reduction.

Few trials were designed to clarify this important issue.

RTOG 9804 and the UK DCIS II trials are both designed to compare RT plus endocrine therapy with endocrine therapy alone for low-risk DCIS (grades 1-2 up to 3 cm with clear margins of at least 3 mm).

RTOG 9804 accrued 636 patients out of a target of 1790, and has been recently closed. The results of these trials will provide further information on the efficacy of excision alone and may allow the development of criteria to identify subgroups of patients who may not require adjuvant RT.

## 5. Ongoing Trials

Although many questions remain open despite the increasing interest in this disease, it is reassuring that there are many ongoing clinical trials to clarify several aspects regarding treatment of DCIS (see http://www.clinicaltrials.gov/ for further information).

The role of MRI in the diagnosis and evaluation of the extent of DCIS will be assessed in several small trials sponsored by the Memorial Sloan-Kettering Cancer Center, the University of California at San Francisco, and in Europe by the Centre Lacassagne in Nice.

Wide excision alone in grades 1-2 DCIS less than 2.5 cm in diameter is being investigated by a phase II trial sponsored by the Dana-Farber Cancer Institute. Similarly, the UK ICR-DCIS-II study is studying adjuvant RT after surgery for hormone-responsive DCIS receiving tamoxifen or anastrozole. This is a randomized trial with a target accrual of 2000 patients started in 2004.

Different radiation approaches are being investigated by several trials. Targeted intraoperative RT (20 Gy to surface of tumor bed) is being evaluated at the USC/Norris Comprehensive Cancer Center in 116 patients, and the estimated completion date is 2013. The role of a boost to the tumor bed is currently studied in a phase III multicentric trial in France, with a projected enrollment of 1950 patients. Hypofractionation is being studied by a randomized trial sponsored by MD Anderson Cancer Center comparing conventional whole-breast radiation to 42 Gy in 16 fractions and a boost of 10 Gy in 4 fractions. Estimated enrolment is 200 patients with an expected completion date by 2014.

NSABP B35 is a randomized trial, with a target of 3100 patients, comparing tamoxifen versus anastrozole in the treatment of postmenopausal women with DCIS; results will be available in 2016.

NSABP B-43 is a randomized trial investigating the role of adjuvant Trastuzumab in women after lumpectomy and RT; completion is estimated in 2019. Finally, neoadjuvat approaches, including the use of Aromatase Inhibitor in hormone-positive DCIS, and of Herceptin in neu + DCIS are currently being investigated.

## 6. Conclusions

Based on available evidence obtained from prospective clinical trials, patients with DCIS have potential benefit from RT after BCS with up to 60% risk reduction for ipsilateral recurrence.

However, the natural history of DCIS after surgical treatment is very variable, and the balance between benefit and risk of RT may differ in patients with low- and high-risk disease.

The ultimate goal in treating DCIS may be to accurately identify which patients can safely omit adjuvant RT because their risk of developing a potential life-threatening relapse with invasive carcinoma is low. Actually, there is no evidence from randomized or prospective trials that it is possible to define a low-risk group of patients for whom RT can be safely avoided.

Finally, although we may identify low-risk patients, the potential impact of LR after conservative treatment of DCIS, and its physical and psychological consequences must be properly and individually considered, as the ultimate goal of treatment may be different for different patients.

Many controversial issues on DCIS will probably be resolved if molecular predictors of progression and relapse to invasive carcinoma can be identified.

## Figures and Tables

**Table 1:** Randomized trials results of excision with or without RT.

Trials	NSABP B-17	EORTC 10853	UKCCR	SWE-DCIS
Patients	818	1010	1030	1046
Date	1985–1990	1986–1996	1990–1998	1987–1999
Median F/U (years)	12	10.2	4.3	5.2
Central path review (%)	76	85	79	20
LR with RT (%)	15.7	15	5.6	7
LR w/o RT (%)	31.7	26	13.6	22

Legend: F/U: followup; LR: local recurrence; w/o: without.
